# Supplementary material for: The evolution of acoustic size exaggeration in terrestrial mammals
Source: Nat Commun. 2016 Sep 6;7:12739. doi: 10.1038/ncomms12739 (PMC5025854; doi:10.1038/ncomms12739)
Supplement: Supplementary Information — Supplementary Tables 1-7 and Supplementary References. [file ncomms12739-s1.pdf]

Supplementary Tables

Supplementary Table 1. Results from the ten best supported PGLS regression models fitted to explain variance in male $\log_{10}$ F0 with male $\log_{10}$ body mass across 67 terrestrial mammal species.

Model	(Intercept)	Log_{10} male body mass	Call type	Habitat	Mating system	df	AICc
BM + λ	4.31	-0.50***				4	120.64
OLS	4.69	-0.54***				4	121.77
OU	4.69	-0.54***				4	121.77
BM + λ	4.28	-0.52***		+		5	123.65
OLS	4.59	-0.56***		+		5	124.00
OU	4.59	-0.56***		+		5	124.00
BM + λ	4.35	-0.49***	+			5	124.48
OLS	4.74	-0.54***	+			5	125.67
OU	4.74	-0.54***	+			5	125.67
BM + λ	4.32	-0.51***	+	+		6	127.67

The PGLS regression models are ordered by AICc value so that the best supported model (with the lowest AICc value) is first in the list. "+" denotes factor included in the model and statistically significant independent variables are indicated with asterisks (* P < 0.05, ** P < 0.01, *** P < 0.001). OLS = non-phylogenetic (ordinary least squares) model, BM + λ = Brownian motion + Pagel's lambda, OU = Ornstein-Uhlenbeck model.

Supplementary Table 2. Results from the ten best supported PGLS regression models fitted to explain variance in male $\log_{10}$ ΔF with male $\log_{10}$ body mass across 35 terrestrial mammal species.

Model	(Intercept)	Log_{10} male body mass	Call type	Habitat	Mating system	df	AICc
BM	4.03	-0.34***		+**		4	-17.76
OU	4.16	-0.36***		+**		5	-15.97
BM	4.00	-0.30***				3	-15.57
BM + λ	4.04	-0.34***		+**		5	-15.40
BM	4.05	-0.33***	+	+**		5	-13.84
OU	4.06	-0.30***				4	-13.48
BM + λ	4.00	-0.30***				4	-13.01
BM + λ	3.95	-0.30***	+*	+**		6	-12.43
OU	4.15	-0.35***	+	+**		6	-11.62
BM + λ	3.81	-0.23***	+*			5	-10.72

The PGLS regression models are ordered by AICc value so that the best supported model (with the lowest AICc value) is first in the list. "+" denotes factor included in the model and statistically significant independent variables are indicated with asterisks (* P < 0.05, ** P < 0.01, *** P < 0.001). BM = Brownian motion, BM + λ = Brownian motion + Pagel's lambda, OU = Ornstein-Uhlenbeck model.

Supplementary Table 3. Results from the ten best supported PGLS regression models fitted to explain variance in male $\log_{10}$ F0 with male size dimorphism across 67 terrestrial mammal species.

Model	(Intercept)	Size dimorphism	Log_{10} male body mass	Call type	Habitat	Mating system	df	AICc
BM + λ	9.26	-4.93	-0.47***				5	116.39
OU	7.69	-2.97	-0.53***				5	119.09
BM + λ	9.51	-5.21	-0.49***		+		6	119.20
BM + λ	9.70	-5.31	-0.45***	+			6	119.94
BM + λ	10.61	-6.45*	-0.46***			+	9	122.24
OU	7.95	-3.17	-0.52***	+			6	122.94
BM + λ	9.87	-5.53	-0.47***	+	+		7	123.01
BM + λ	11.01	-6.89*	-0.49***		+	+	10	124.57
BM + ϱ	10.23	-5.66	-0.49***				5	125.20
OU	8.24	-3.56	-0.54***			+	9	125.37

The PGLS regression models are ordered by AICc value so that the best supported model (with the lowest AICc value) is first in the list. "+" denotes factor included in the model and statistically significant independent variables are indicated with asterisks (* P < 0.05, ** P < 0.01, *** P < 0.001). BM + λ = Brownian motion + Pagel's lambda, BM + ϱ = Brownian motion + Grafen's rho, OU = Ornstein-Uhlenbeck model.

Supplementary Table 4. Results from the ten best supported PGLS regression models fitted to explain variance in male $\log_{10}$ ΔF with male size dimorphism across 35 terrestrial mammal species.

Model	(Intercept)	Size dimorphism	Log_{10} male body mass	Call type	Habitat	Mating system	df	AICc
BM	7.58	-3.58**	-0.31***		+**		5	-25.22
BM + λ	8.27	-4.25**	-0.31***		+**		6	-23.75
OU	7.90	-3.80**	-0.32***		+**		6	-23.19
BM	7.82	-3.86**	-0.26***				4	-23.05
OU	8.23	-4.17**	-0.27***				5	-21.03
BM	7.45	-3.44**	-0.30***	+	+**		6	-20.90
BM + λ	8.18	-4.20**	-0.26***				5	-20.61
OU	7.71	-3.61**	-0.31***	+	+**		7	-18.35
BM + λ	8.06	-4.03**	-0.30***	+	+**		7	-18.29
BM	7.74	-3.77**	-0.25***	+			5	-17.48

The PGLS regression models are ordered by AICc value so that the best supported model (with the lowest AICc value) is first in the list. "+" denotes factor included in the model and statistically significant independent variables are indicated with asterisks (* P < 0.05, ** P < 0.01, *** P < 0.001). BM = Brownian motion, BM + λ = Brownian motion + Pagel's lambda, OU = Ornstein-Uhlenbeck model.

Supplementary Table 5. Results from the ten best supported PGLS regression models fitted to explain variance in male $\log_{10}$ F0 with $\log_{10}$ male relative testes size across 42 terrestrial mammal species.

Model	(Intercept)	Log_{10} relative testes size	Log_{10} male body mass	Call type	Habitat	Mating system	df	AICc
OU	4.45	0.39*	-0.42***				5	86.24
BM	3.93	0.33*	-0.36***				4	86.84
BM + λ	4.15	0.33*	-0.41***				5	87.16
OU	4.41	0.36*	-0.45***		+		6	88.88
BM	3.90	0.31*	-0.37**		+		5	89.86
BM + λ	4.12	0.31	-0.42***		+		6	90.32
OU	4.49	0.40*	-0.41***	+			6	90.34
OLS	4.65	0.48*	-0.44***				4	90.44
BM	3.93	0.33*	-0.35***	+			5	91.05
BM + ϱ	4.46	0.43*	-0.42***				5	91.06

The PGLS regression models are ordered by AICc value so that the best supported model (with the lowest AICc value) is first in the list. "+" denotes factor included in the model and statistically significant independent variables are indicated with asterisks (* P < 0.05, ** P < 0.01, *** P < 0.001). OLS = non-phylogenetic (ordinary least squares) model, BM = Brownian motion, BM + λ = Brownian motion + Pagel's lambda, BM + ϱ = Brownian motion + Grafen's rho, OU = Ornstein-Uhlenbeck model.

Supplementary Table 6. Results from the ten best supported PGLS regression models fitted to explain variance in male $\log_{10}$ ΔF with male relative testes size across 24 terrestrial mammal species.

Model	(Intercept)	Log_{10} relative testes size	Log_{10} male body mass	Call type	Habitat	Mating system	df	AICc
BM + λ	4.12	0.10***	-0.31***				5	-6.70
BM	4.10	0.10**	-0.31***				4	-5.78
BM + λ	4.15	0.10**	-0.34***		+		6	-3.75
BM	4.15	0.09**	-0.34***		+		5	-3.65
OU	4.10	0.10**	-0.31***				5	-2.55
BM + ϱ	4.14	0.11**	-0.32***				5	-2.27
BM + ϱ	4.06	0.09**	-0.33***		+		6	0.57
BM + λ	4.11	0.11**	-0.31***	+			6	1.18
BM	4.11	0.10**	-0.31***	+			5	1.29
BM	4.15	0.09**	-0.34***	+	+		6	3.94

The PGLS regression models are ordered by AICc value so that the best supported model (with the lowest AICc value) is first in the list. "+" denotes factor included in the model and statistically significant independent variables are indicated with asterisks (* P < 0.05, ** P < 0.01, *** P < 0.001). BM = Brownian motion, BM + λ = Brownian motion + Pagel's lambda, BM + ϱ = Brownian motion + Grafen's rho, OU = Ornstein-Uhlenbeck model.

Supplementary Table 7. Acoustic, morphological and ecological data for each of the terrestrial mammal species in the dataset

Species	Habitat	Mating system	Male body mass (g)	Size Dimorphism	Male body mass for testes data (g)	Testes mass (g)	Mean F0 (Hz)	Mean ΔF (Hz)	References
Ailuropoda melanoleuca	Terrestrial	PO	117900	1.02	117180	274.7*	372	555	1,2
Alces alces	Terrestrial	PO	323000	1.02	789000	106.0	125	-	3-6
Alopex lagopus	Terrestrial	MO	3500	1.02	4800	4.1	990	-	7-9
Alouatta caraya	Arboreal	PR	6400	1.05	64000	17.0*	50	535	5,10
Alouatta guariba	Arboreal	PO	6700	1.04	67000	8.5*	-	478	10
Alouatta macconnellii	Arboreal	PO	7600	1.05	-	-	-	393	10
Alouatta palliata	Arboreal	PO	5800	1.03	7260	23.0	-	581	4,5,10
Alouatta pigra	Arboreal	PO	7600	1.07	76000	11.5*	-	445	10,11
Alouatta seniculus	Arboreal	PO	6700	1.04	67000	3.6*	-	418	5,10
Bison bison	Terrestrial	PO	863410	1.04	299400	439.8	55	317	5,12-14
Brachyteles hypoxanthus	Arboreal	PR	9600	1.01	-	-	1731	-	15
Callithrix jacchus	Arboreal	MO	369	1.01	320	1.3	7435	-	4,15
Capreolus capreolus	Terrestrial	PO	23025	1.01	20500	45.0	200	760	5,6,9
Cebus capucinus	Arboreal	PR	3626	1.05	-	-	731	-	15
Cercocebus atys	Arboreal	PR	10553	1.06	-	-	77	-	15
Cercopithecus diana	Arboreal	PO	8700	1.03	-	-	60	900	11,16,17
Cercopithecus mona	Arboreal	PO	4566	1.06	-	-	334	-	15
Cercopithecus nictitans	Arboreal	PO	6633	1.05	-	-	220	629	4,18
Cervus elaphus canadensis	Terrestrial	PO	289750	1.02	-	-	145	222	5,19
Cervus elaphus corsicanus	Terrestrial	PO	88000	1.03	-	-	40	256	3,20
Cervus elaphus scoticus	Terrestrial	PO	125000	1.04	122000	218.0	112	247	4,5,21
Cervus nippon	Terrestrial	PO	39450	1.04	-	-	933^	-	5,22
Colobus guereza	Arboreal	PO	13390	1.04	10400	3.0	18^	717	11,23,24
Cryptomys hottentotus	Terrestrial	PA	90	1.06	104	0.6	2050^	-	8,9,25
Cryptomys mechowi	Terrestrial	PA	350	1.07	-	-	500	-	8,25
Dama dama	Terrestrial	PO	59166	1.02	63700	133.0	34	280	3-5,26
Elephas maximus	Terrestrial	PO	4650000	1.03	4545000	4000	60	-	4,8,27
Eptesicus fuscus	Arboreal	PO	16	0.95	-	-	16800^	-	5,28
Erythrocebus patas	Terrestrial	PO	9740	1.08	10000	7.2	150	-	15
Eulemur fulvus	Arboreal	VA	1970.00	0.99	2500	7.8	566	2532	4,9,11,29
Eulemur macaco	Arboreal	PO	2370.00	0.99	2500	16.7	305	2567	11,23,29
Eulemur rubriventer	Arboreal	MO	1980	1.00	2512	0.9	48	1545	11,30,31
Felis margarita	Terrestrial	PO	3000	1.02	-	-	360	-	4,32
Felis nigripes	Terrestrial	PO	1800	1.04	1983	2.0	310	-	2,4,32
Felis silvestris lybica	Terrestrial	PO	5000	1.02	4528	2.6	362	-	2,4,32
Felis silvestris ornata	Terrestrial	PO	3900	1.05	-	-	260	-	32
Felis silvestris silvestris	Terrestrial	PO	7100	1.05	4600	1.4	300	-	2,4,32
Gazella subgutturosa	Terrestrial	PO	25500	1.04	-	-	45	382	5,33
Gorilla gorilla	Terrestrial	PO	154000	1.07	168250	26.7	166	-	4,23,34
Heterocephalus glaber	Terrestrial	PA	30	1.00	-	-	3000^	-	8,25
Homo sapiens	Terrestrial	VA	74960	1.02	65650	40.5	114	1011	4,35,36
Hylobates lar	Arboreal	MO	5900	1.01	5500	5.5	1076	-	4,11,15,37
Hypsignathus monstrosus	Arboreal	PO	333	1.07	354	0.2	290	-	4,8,38
Indri indri	Arboreal	MO	5830	0.98	-	-	653	-	11,39
Loxodonta africana	Terrestrial	PO	5374000	1.05	4365000	4530	14	51	4,8,40,41
Macaca mulatta	Terrestrial	PR	7506	1.03	9200	46.2	160	1421	4,8,42,43
Marmota flaviventris	Terrestrial	PO	5067	1.05	-	-	1275	-	8,44
Meles meles	Terrestrial	PR	10120	1.02	14515	14.4	14	-	4,5,45
Mesocricetus auratus	Terrestrial	PO	104	1.06	108	3.2	34500	-	4,8,46
Microcebus murinus	Arboreal	PO	78	1.01	70	2.5	23700	-	8,23,47
Microcebus rufus	Arboreal	PO	43	1.01	-	-	21800^	-	11,48
Mus musculus	Terrestrial	PO	15	0.99	15	0.1	62775	-	4,5,49
Odocoileus virginianus	Terrestrial	PO	76616	1.03	71000	76.0	209	-	3-5
Ovibos moschatus	Terrestrial	PO	398000	1.02	-	-	89	360	8,50
Pan troglodytes	Terrestrial	PR	56668	1.02	45000	139.0	718	1122	8,23,51,52
Panthera leo	Terrestrial	PO	189000	1.02	188000	55.0	195	308	4,5,53
Panthera tigris	Terrestrial	PR	182000	1.04	-	-	29	-	5,54
Papio hamadryas	Terrestrial	PO	29800	1.06	26400	93.5	52	998	4,8,55
Papio ursinus	Terrestrial	PO	31050	1.07	-	-	53	738	5,56
Phascolarctos cinereus	Arboreal	PO	7700	1.03	11100	15.0	27	354	4,5,57
Phodopus sungorus	Terrestrial	PO	37	1.05	42	0.9	57600	-	4,8,58
Pongo pygmaeus	Arboreal	PO	74700	1.06	74640	35.30	412^	-	4,5,59
Procapra gutturosa	Terrestrial	PO	31500	1.03	-	-	500	564	4,60
Procavia capensis	Terrestrial	PO	2738	1.00	-	-	1280	2766	5,61
Rangifer tarandus	Terrestrial	PO	172000	1.04	145000	132.0	55	-	3-5
Rattus rattus	Terrestrial	PO	133	1.01	169	7.2	48916	-	4,62,63
Saiga tatarica	Terrestrial	PO	41750	1.03	-	-	45	407	5,64
Spalax ehrenbergi	Terrestrial	MO	170	1.03	-	-	550	-	8,25
Suricata suricatta	Terrestrial	MO	760	1.01	731	1.3	566	2394	4,9,65,66
Theropithecus gelada	Terrestrial	PO	20500	1.51	20400	17.10	111	1051#	4,5,67
Tupaia belangeri	Terrestrial	MO	229	1.04	-	-	7200^	-	68,69
Varecia variegata	Arboreal	VA	3630	1.03	4750	17.17	222	1464	4,11,29,70

PO = Polygynous, MO = Monogamous, PR = Promiscuous, PA = Polyandrous, VA = Variable.
Data on species habitat were taken from the Encyclopedia of Life website (http://eol.org/)
Data on species mating system were taken from the Animal Diversity Website (http://animaldiversity.org/)
* Testes mass in grams was calculated by multiplying the volume in mm3 by 1.02 [2].
^ F0 calculated as the mid point between reported minimum and maximum values.
# Formant frequency values measured from a published spectrogram using a clear ruler to extrapolate from the axes.

Supplementary References

1 Charlton, B., Zhang, Z. & Snyder, R. Vocal cues to identity and relatedness in giant pandas (*Ailuropoda melanoleuca*). *J. Acoust. Soc. Am.* **126**, 2721-2732 (2009).
2 Iossa, G., Soulsbury, C. D., Baker, P. J. & Harris, S. Sperm competition and the evolution of testes size in terrestrial mammalian carnivores. *Funct. Ecology* **22**, 655-662 (2008).
3 Cap, H., Deleporte, P., Joachim, J. & Reby, D. Male vocal behavior and phylogeny in deer. *Cladistics* **24**, 917-931 (2008).
4 Morrow, E. H. & Fricke, C. Sexual selection and the risk of extinction in mammals. *Proc. Biol. Sci.* **271**, 2395-2401 (2004).
5 Silva, M. & Downing, J. A. *CRC handbook of mammalian body masses*. (CRC press, 1995).
6 Reby, D. & Cargnelutti, B. ''Des Voix Dans la Foreˆt. Un Guide Sonore des Cervides d'Europe.'' Sittelle, Mens. (1999).
7 Frommolt, K. H., Goltsman, M. E. & MacDonald, D. W. Barking foxes, Alopex lagopus: field experiments in individual recognition in a territorial mammal. *Anim. Behav.* **65**, 509-518 (2003).
8 Jones, K. E. *et al.* PanTHERIA: a species-level database of life history, ecology, and geography of extant and recently extinct mammals. *Ecology* **90** 2648-2648 (2009).
9 Soulsbury, C. D. Genetic patterns of paternity and testes size in mammals. *PLoS ONE* **5**, e9581-9587 (2010).
10 Dunn, J. C. *et al.* Evolutionary Trade-Off between Vocal Tract and Testes Dimensions in Howler Monkeys. *Curr Biol* **25**, 2839-2844 (2015).
11 Smith, R. J. & Junkers, W. L. Body mass in comparative primatology. *Journal of Human Evolution* **32**, 523-559 (1997).
12 Helbig, L., Woodbury, M. R., Haigh, J. C., Collins, J. & Barth, A. D. The seasonal fertility of North American bison (*Bison bison*) bulls. *Anim. Reprod. Sci.* **97**, 265-277.
13 Wyman, M. T. (Unpublished).
14 Wyman, M. T. *et al.* Acoustic cues to size and quality in the vocalizations of male North American bison, *Bison bison*. *Anim. Behav.* **84**, 1381-1391 (2012).
15 Puts, D. A. *et al.* Sexual selection on male vocal fundamental frequency in humans and other anthropoids. *Proc. Biol. Sci.* **283** (2016).
16 Riede, T. & Zuberbühler, K. Pulse register phonation in Diana monkey alarm calls. *J. Acoust. Soc. Am.* **113**, 2919-2926 (2003).
17 Riede, T., Bronson, E., Hatzikirou, H. & Zuberbuhler, K. Vocal production mechanisms in a non-human primate: morphological data and a model. *J. Hum. Evol.* **48**, 85-96 (2005).
18 Price, T., Arnold, K., Zuberbühler, K. & Semple, S. Pyow but not hack calls of the male putty-nosed monkey (*Cercopithcus nictitans*) convey information about caller identity. *Behaviour* **146**, 871-888 (2009).
19 Reby, D. *et al.* Evidence of biphonation and source-filter interactions in the bugles of male North American elk (*Cervus canadensis*). *J. Exp. Biol.* **In press** (2016).
20 Kidjo, N., Cargnelutti, B., Charlton, B. D., Wilson, C. & Reby, D. Vocal behaviour in the endangered Corsican deer, description and phylogenetic implications. *Bioacoustics* **18**, 159-181 (2008).
21 Reby, D. & McComb, K. Anatomical constraints generate honesty: acoustic cues to age and weight in the roars of red deer stags. *Anim. Behav.* **65**, 519-530 (2003).
22 Minami, M. & Kawamichi, T. Vocal repertoires and classification of the sika deer *Cervus nippon*. *J. Mamm. Soc. Japan* **17**, 71-94 (1992).
23 Harcourt, A. H., Purvis, A. & Liles, L. Sperm competition: mating system, not breeding season, affects testes size of primates. *Funct. Ecol.* **9**, 468-476 (1995).
24 Harris, T. R., Fitch, W. T., Goldstein, L. M. & Fashing, P. J. Black and white colobus monkey (*Colobus guereza*) roars as a source of both honest and exaggerated information about body mass. *Ethology* **112**, 911-920 (2006).
25 Credner, S., Burda, H. & Ludescher, F. Acoustic communication underground: vocalization characteristics in subterranean social mole-rats (*Cryptomys* sp., Bathyergidae). *J Comp. Physiol. A: Neuroethology* **180**, 245-255 (1997).
26 McElligott, A. G., Birrer, M. & Vannoni, E. Retraction of the mobile descended larynx during groaning enables fallow bucks (*Dama dama*) to lower their formant frequencies. *J. Zool.* **270**, 340-345 (2006).
27 De Silva, S. Acoustic communication in the Asian elephant, *Elephas maximus maximus*. *Behaviour* **147**, 825-852 (2010).
28 Gadziola, M. A., Grimsley, J. M. S., Faure, P. A. & Wenstrup, J. J. Social vocalizations of big brown bats vary with behavioral context. *PLoS ONE* **7**, e44550-44516 (2012).
29 Gamba, M. (Unpublished).
30 Kappeler, P. M. Intrasexual selection and testis size in strepsirhine primates. *Behav. Ecol.* **8**, 10-19 (1997).
31 Gamba, M., Colombo, C. & Giacoma, C. Acoustic cues to caller identity in lemurs: a case study. *J. Ethol.* **30**, 191-196 (2012).
32 Peters, G., Baum, L., Peters, M. & Tonkin-Leyhausen, B. Spectral characteristics of intense mew calls in cat species of the genus Felis (*Mammalia: Carnivora: Felidae)*. *J. Ethol.* **27**, 221-237 (2009).
33 Frey, R., Volodin, I., Volodina, E., Soldatova, N. V. & Juldaschev, E. T. Descended and mobile larynx, vocal tract elongation and rutting roars in male goitred gazelles (Gazella subgutturosa Güldenstaedt, 1780). *J. Anat.* **218**, 566-585 (2011).
34 Salmi, R. & Doran-Sheehy, D. M. The function of loud calls (Hoot Series) in wild western gorillas (*Gorilla gorilla*). *Am. J. Phys. Anthropol.* **155**, 379-391 (2014).
35 Gage, M. J. G. & Freckleton, R. P. Relative testis size and sperm morphometry across mammals: no evidence for an association between sperm competition and sperm length. *Proc. Biol. Sci.* **270**, 625-632 (2003).
36 Pisanski, K. *et al.* Voice parameters predict sex-specific body morphology in men and women. *Anim. Behav.* **112**, 13-22 (2016).
37 Barelli, C., Mundry, R., Heistermann, M. & Hammerschmidt, K. Cues to androgens and quality in male gibbon songs. *PLoS ONE* **8**, e82748 (2013).
38 Bradbury, J. Lek mating behavior in the hammer–headed bat. *Zeits. Tierpsychol.* **45**, 225-255 (1977).
39 Giacoma, C., Sorrentino, V., Rabarivola, C. & Gamba, M. Sex differences in the song of *Indri indri*. *Int. J. Primatol.* **31**, 539-551 (2010).
40 Poole, J. H., Payne, K., Langbauer, W. R. & Moss, C. J. The social contexts of some very low frequency calls of African elephants. *Behav. Ecol. Sociobiol.* **22**, 385-392 (1988).
41 Stoeger, A. S. & Baotic, A. Information content and acoustic structure of male African elephant social rumbles. *Sci. Rep.* **6**, 1-8 (2016).
42 Fitch, W. T. Vocal tract length and formant frequency dispersion correlate with body size in rhesus macaques. *J. Acoust. Soc. Am.* **102**, 1213-1222 (1997).
43 Rendall, D., Owren, M. J. & Rodman, P. S. The role of vocal tract filtering in identity cueing in rhesus monkey (Macaca mulatta) vocalizations. *J. Acoust. Soc. Am.* **103**, 602-614 (1998).
44 Blumstein, D. & Munos, O. Individual, age and sex-specific information is contained in yellow-bellied marmot alarm calls. *Anim. Behav.* (2005).
45 Wong, J., Stewart, P. D. & Macdonald, D. W. Vocal repertoire in the European badger (*Meles meles*): Structure, context, and function. *J. Mammal.* **80**, 570-588 (1999).
46 Floody, O. R. & Pfaff, D. W. Communication among hamsters by high-frequency acoustic signals: I. Physical characteristics of hamster calls. *J. Comp. Physiol. Psychol.* **91**, 794-806 (1977).
47 Zimmerman, E. & Lerch, C. The complex acoustic design of an advertisement call in male mouse lemurs (*Microcebus murinus*) and sources of its variation. *Ethology* **93**, 211-224 (1993).
48 Zimmermann, E., Vorobieva, E., Wrogemann, D. & Hafen, T. Use of vocal fingerprinting for specific discrimination of gray (*Microcebus murinus*) and rufous mouse lemurs (*Microcebus rufus*). *Int. J. Primatol.* **21**, 837-852 (2000).
49 Gourbal, B. E. F., Barthelemy, M., Petit, G. & Gabrion, C. Spectrographic analysis of the ultrasonic vocalisations of adult male and female BALB/c mice. *Naturwissenschaften* **91**, 1-5 (2004).
50 Frey, R., Gebler, A. & Fritsch, G. Arctic roars - laryngeal anatomy and vocalization of the muskox (*Ovibos moschatus* Zimmermann, 1780, *Bovidae*). *J. Zool.* **268**, 433-448 (2006).
51 Riede, T., Arcadi, A. C. & Owren, M. J. Nonlinear acoustics in the pant hoots of common chimpanzees (*Pan troglodytes*): Vocalizing at the edge. *J. Acoust. Soc. Am.* **121**, 1758-1767 (2007).
52 Slocombe, K. E. & Zuberbuhler, K. Functionally referential communication in a chimpanzee. *Curr. Biol.* **15**, 1779-1784 (2005).
53 Pfefferle, D., West, P. M., Grinnell, J., Packer, C. & Fischer, J. Do acoustic features of lion, *Panthera leo*, roars reflect sex and male condition? *J. Acoust. Soc. Am.* **121**, 3947-3953 (2007).
54 Titze, I. R. *et al.* Vocal power and pressure-flow relationships in excised tiger larynges. *J. Exp. Biol.* **213**, 3866-3873 (2010).
55 Pfefferle, D. & Fischer, J. Sounds and size: identification of acoustic variables that reflect body size in hamadryas baboons, *Papio hamadryas*. *Anim. Behav.* **72**, 43-51 (2006).
56 Rendall, D., Owren, M., Weerts, E. & Hienz, R. Sex differences in the acoustic structure of vowel-like grunt vocalizations in baboons and their perceptual discrimination by baboon listeners. *J. Acoust. Soc. Am.* (2003).
57 Charlton, B. *et al.* Cues to body size in the formant spacing of male koala (Phascolarctos cinereus) bellows: honesty in an exaggerated trait. *J. Exp. Biol.* **214**, 3414-3422, doi:10.1242/jeb.061358 (2011).
58 Keesom, S. M., Rendon, N. M., Demas, G. E. & Hurley, L. M. Vocal behaviour during aggressive encounters between Siberian hamsters, *Phodopus sungorus*. *Anim. Behav.* **102**, 85-93 (2015).
59 Spillmann, B. *et al.* Acoustic properties of long calls given by flanged male orang-utans (*Pongo pygmaeus wurmbii*) reflect both individual identity and context. *Ethology* **116**, 385-395 (2010).
60 Frey, R. & Gebler, A. The highly specialized vocal tract of the male Mongolian gazelle (*Procapra gutturosa* Pallas, 1777 - *Mammalia*, *Bovidae*). *J. Anat.* **203**, 451-471 (2003).
61 Koren, L. & Geffen, E. Complex call in male rock hyrax (*Procavia capensis*): a multi-information distributing channel. *Behav. Ecol. Sociobiol.* **63**, 581-590 (2009).
62 Haney, M. & Miczek, K. A. Ultrasounds during agonistic interactions between female rats (*Rattus norvegicus*). *J. Comp. Psychol.* **107**, 373-379 (1993).
63 White, N. R., Cagiano, R., Moises, A. U. & Barfield, R. J. Changes in mating vocalizations over the ejaculatory series in rats (*Rattus norvegicus*). *J. Comp. Psychol.* **104**, 255-262 (1990).
64 Frey, R., Volodin, I. & Volodina, E. A nose that roars: anatomical specializations and behavioural features of rutting male saiga. *J. Anat.* **211**, 717-736 (2007).
65 Townsend, S. W., Zöttl, M. & Manser, M. B. All clear? Meerkats attend to contextual information in close calls to coordinate vigilance. *Behav. Ecol. Sociobiol.* **65**, 1927-1934 (2011).
66 Townsend, S., Charlton, B. & Manser, M. Acoustic cues to identity and predator context in meerkat barks. *Anim. Behav.* **94**, 143-149, (2014).
67 Bergman, T. J. Speech-like vocalized lip-smacking in geladas. *Curr. Biol.* **23**, R268-R269 (2013).
68 Schehka, S. & Zimmermann, E. Acoustic features to arousal and identity in disturbance calls of tree shrews (Tupaia belangeri). *Behav. Brain Res.* **203**, 223-231 (2009).
69 Binz, H. & Zimmermann, E. The vocal repertoire of adult tree shrews (*Tupaia belangeri*). *Behaviour* **109**, 142-162 (1989).
70 Pereira, M. E., Seeligson, M. I. & Macedonia, J. The behavioral repertoire of the black-and-white ruffed lemur, *Varecia variegata variegata* (Primates: Lemuridae). *Folia Primatol.* **51**, 1-32 (1988).
